# Effectiveness of a real-life program (DIAfit) to promote physical activity in patients with type 2 diabetes: a pragmatic cluster randomized clinical trial

**DOI:** 10.3389/fendo.2023.1155217

**Published:** 2023-07-03

**Authors:** Amar Arhab, Nicolas Junod, Jean-Benoit Rossel, Olivier Giet, Frederic Sittarame, Sandra Beer, Daniela Sofra, Dominique Durrer, Humberto Delgado, Montserrat Castellsague, Markus Laimer, Jardena J. Puder

**Affiliations:** ^1^ Obstetric Service, Department Woman-Mother-Child, Lausanne University Hospital, Centre Hospitalier Universitaire Vaudois (CHUV), Lausanne, Lausanne, Switzerland; ^2^ La Lignière Clinic, Department of Medicine, Cardiovascular and Metabolic Rehabilitation Service, Gland, Switzerland; ^3^ Lavaux Hospital, Cully, Switzerland; ^4^ Socio-medical Centre, Sierre, Switzerland; ^5^ Cardiac Rehabilitation and Heart Failure Unity, Cardiology Service, Department of Medicine, University Hospitals Geneva, Geneva, Switzerland; ^6^ Private Practice for Diabetes and Endocrinology, Lausanne, Switzerland; ^7^ European Association for the Study of Obesity (EASO), Collaborating Centre for Obesity Management (COMs), Vevey, Switzerland; ^8^ Direction of Nursing Care, University Hospitals Geneva, Geneva, Switzerland; ^9^ Department of Diabetes, Endocrinology, Nutritional Medicine and Metabolism, Inselspital Bern University Hospital, Bern, Switzerland

**Keywords:** type 2 diabetes mellitus, aerobic fitness, body composition, physical activity, randomized clinical trial

## Abstract

**Introduction:**

The aim of this study was to evaluate the effectiveness of a real-life clinical physical activity program (DIAfit) on improving physical fitness, body composition, and cardiometabolic health in an unselected population with type 2 diabetes mellitus, and to compare the effects of two variants a different exercise frequencies on the same outcomes.

**Research design and methods:**

This was a cluster randomized-controlled assessor-blind trial conducted in 11 clinical centres in Switzerland. All participants in the clinical program with type 2 diabetes were eligible and were randomized to either standard (3 sessions/week for 12 weeks) or alternative (1 session/week for the first four weeks, then 2 sessions/week for the rest of 16 weeks) physical activity program each consisting of 36 sessions of combined aerobic and resistance exercise. Allocation was concealed by a central office unrelated to the study. The primary outcome was aerobic fitness. Secondary outcome measures included: body composition, BMI, HbA_1c_, muscle strength, walking speed, balance, flexibility, blood pressure, lipid profile.

**Results:**

All 185 patients with type 2 diabetes (mean age 59.7 +-10.2 years, 48% women) agreed to participate and were randomized in two groups: a standard group (n=88) and an alternative group (n=97)). There was an 11% increase in aerobic fitness after the program (12.5 Watts; 95% CI 6.76 to 18.25; p<0.001). Significant improvements in physical fitness, body composition, and cardiometabolic parameters were observed at the end of the DIAfit program (improvements between 2-29%) except for lean body mass, triglycerides and cholesterol. No differences were observed between both programs, except for a larger weight reduction of -0.97kg (95% CI -0.04 to -1.91; p=0.04) in the standard program.

**Conclusions:**

Both frequency variants of the nation-wide DIAfit program had beneficial effects on physical fitness, HbA_1c_, body composition, and blood pressure in type 2 diabetes patients and differences were negligible.

**Clinical trial registration:**

clinicaltrials.gov, identifier NCT01289587.

## 1 Introduction

The global prevalence of type 2 diabetes is high (8.8%) and is projected to increase considerably in the next years (1). Physical activity (PA) is a cornerstone of diabetes management and overall health (2, 3). International guidelines for the general population and patients with type 2 diabetes recommend a minimum of 150 min/week of moderate-intensity aerobic physical activity and at least 2 times per week resistance training (4–6). In addition, flexibility and balance training are recommended for older adults with type 2 diabetes (7). Structured PA training programs have been performed and shown to be effective in improving glycaemic control, aerobic fitness and reducing other cardiovascular risk factors in patients with type 2 diabetes (8–11). Although the efficacy of intensive PA interventions in specialized research and clinical centres has been proven, an important challenge remains in translating PA interventions into pragmatic programs in real-life settings. Moreover, there does not seem to be an effective “one size fits all” approach to engage the general population with diabetes in increasing PA.

A Cochrane review has stated that the optimal type, frequency, intensity and duration of exercise for achieving therapeutic goals in type 2 diabetes patients are still unknown (3). This was further corroborated by a more recent meta-analysis and meta-regression that showed there is still insufficient evidence on the exact intensity, volume and duration of exercise required to provide optimal glycaemic control (12). Most available studies have been performed using a structure of three sessions per week. A meta-analysis of randomized controlled trials showed that most of the PA interventions consisted of at least three structured exercise sessions per week (8), and it showed improvements in HbA_1c_ or physical fitness measures (13). Three or more sessions per week are not always feasible for some type 2 diabetes patients. The optimal PA frequency to improve aerobic fitness, muscular fitness, body composition, and metabolic parameters and to also provide sustainable adherence is not clear (13–15). Few studies have examined the influence of two or less weekly supervised training sessions on the management of type 2 diabetes, and the results are inconsistent (16–20). Some showed improvements in HbA_1c_ and lipid profile (16, 18, 19), whereas others did not observe such improvements (17, 20). Moreover, two of these studies showed additional improvements in aerobic fitness, muscular strength, BP, cholesterol level, waist circumference and BMI (16, 18). However, they were conducted in specialized centres. Importantly, they offered only one type of exercise frequency and did not compare different options. To our knowledge, no recent studies looked at the impact of exercise frequency on aerobic fitness, strength, flexibility, balance and walking speed, body composition and cardiometabolic health in type 2 diabetes patients. Hence, more translational studies are needed to compare alternatives strategies of exercise training focusing on frequency to tailor a pragmatic exercise program as part of standard clinical practice in patients with type 2 diabetes.

This pragmatic randomized trial investigated the feasibility and overall effectiveness of a nationwide PA program in a real-life clinical setting in patients with type 2 diabetes. Firstly, we evaluated the effects of the DIAfit program on aerobic fitness (primary outcome), as well as on strength, flexibility, balance and walking speed, anthropometry, body composition and cardiometabolic parameters independent of the selected frequency variant of the program. Secondly, we compared the effectiveness of the standard DIAfit program (3x/week over 12 weeks) with an alternative program including the same total sessions but differed in frequency (increasing frequency from 1x/week up to 2x/week over 20 weeks) on the same outcomes.

## 2 Materials and methods

### 2.1 Design, setting and participants

The clinical DIAfit program is a nationwide Swiss lifestyle based real-life clinical group intervention program (www.diafit.ch). Recruitment took place between September 2010 and December 2011. It was simultaneously introduced in 2011 in 11 diverse treatment centres in the French-speaking part of Switzerland. The basic health insurance approved the reimbursement of the program under the following criteria: presence of an adapted infrastructure, the ability to provide CPR, a diabetologist that had undergone a one-day training and a physical therapist/sport scientist that had undergone a 2-week training. To ensure quality of delivery, each centre had to provide an entry and exit medical visit, offer 36 supervised exercise sessions that included both resistance and endurance activities and ideally also balance and flexibility training as well as 6-8 educational group sessions about diabetes and lifestyle. Eligibility criteria for the treatment centres to participate in the study was defined by the ability to offer the clinical program. All 11 centres eligible to participate in the clinical program underwent simultaneous training and agreed to participate in the study and none of the centres dropped out during the study. Centres included 7 small regional hospitals or medical centres, 2 private practices and 2 tertiary university hospitals. All 11 centres had to fulfil the following conditions: to provide a trained a responsible physician that oversees the program and carries the medical responsibilities, a qualified physical therapist, a qualified diabetologist who is also member of the Swiss Society of Endocrinology and Diabetes who has a consultant role, and a qualified dietician.

Local diabetologist or general practitioners referred their patients to the closest clinical program centre. Inclusion criteria for patient to be enrolled in the clinical DIAfit program were age ≥18 years and a diagnosis of type 2 diabetes for ≥3 months. Exclusion criteria for clinical referrals were active diabetic foot ulceration; ischemia during a cycle ergometry exercise test or state III peripheral vascular disease; untreated proliferative retinopathy and autonomic neuropathy with unstable BP during the exercise test. In order to increase external validity, all patients referred to the clinical program were eligible to participate in the study. All 185 eligible and participating patients that were referred to the clinical program between 2011 and 2012 from the 11 DIAfit treatment centres were invited to participate in the study.

All patients underwent the same medical visit by physicians collaborating in the DIAfit program. As part of the clinical routine, patients had a fasting blood analysis and a cycle ergometry according to a standardized protocol at the beginning and the end. For the study, they underwent additional physical fitness tests and filled out questionnaires at both time points. During the centralized clinical training all centres were instructed how to perform all physical fitness tests and medical exams according to standard operating procedures. Baseline data of this study have been previously reported (21).

### 2.2 Randomization and blinding

DIAfit was a cluster randomized controlled single-blind trial 1:1. At the start of the clinical program, two different exercise frequency variants were introduced and tested. Patient groups were allocated per cluster (i.e., treatment centre) to randomly start one program variant first and then the other using concealed opaque envelopes. Each centre thus provided both variants. Selection and randomization took place 2 weeks before the start of each program and were performed by a central coordinator (OG) who was not involved in the study. Patients signed informed consent before knowing their group allocation. Health care providers and investigators were blinded to group allocation before the start of the first program. The primary outcome assessors were always blinded to the respective variant. Patients and the clinical staff such as the physical therapists could not be blinded since they knew how many sessions per week were attended. Both program variants were matched by number of sessions (22) but differed by the frequency of the sessions. Participants in the standard program, as a control group, performed 36 supervised sessions over 12 weeks (3 sessions/week), whereas the alternative program performed 36 supervised sessions over 20 weeks (1 session/week first month, then 2 sessions/week). The rationale behind the standard program was the classical frequency used in most studies and the physiological approach (not more than 2 days without PA, due to the dynamic of its effects on insulin resistance) (13), while the rationale behind the alternative program was a feasibility approach (time limitations, difficulty for sedentary patients to suddenly participate 3x/week in PA sessions, severe deconditioning making a stepwise increase in frequency necessary). Both programs had the same content (both endurance and resistance training) and concomitantly encouraged unstructured PA (particularly walking and taking stairs) in the daily life. The study was designed to intervene at the individual level. See **Table 1** for more details of interventions.

**Table 1:** Details of the standard and alternative interventions.

	Standard program	Alternative program
Frequency	3 sessions/week	1 session/week for 4 weeks, then 2 sessions/week
Session duration	50-60min	50-60min
Program duration	12 weeks (36 sessions)	20 weeks (36 sessions)
Intensity		
*Aerobic exercise*	70-85% of maximal HR	70-85% of maximal HR
*Resistance exercise*	3 sets of 10 repetitions with 60-80% of 1 RM	3 sets of 10 repetitions with 60-80% of 1 RM
Type/Mode		
*Aerobic exercise*	Bicycle ergometer	Bicycle ergometer
*Resistance exercise*	6 exercises for large muscle groups (2 legs: extension/flexion, 2 upper-body: extension/flexion, 2 abdominals and back)	6 exercises for large muscle groups (2 legs: extension/flexion, 2 upper-body: extension/flexion, 2 abdominals and back)
Both interventions		
Before exercise	HR, BP, and glucose level	
Warm-up	10min	
Workshops	6-8 workshops on promoting positive lifestyle behaviour change	

HR, heart rate; RM, repetition maximum.

### 2.3 Ethics

The study was approved by the ethical committee of Lausanne in 2011 (protocol 252/10) and all participants signed a written informed consent. The trial was registered (clinicaltrials.gov NCT01289587).

### 2.4 Outcomes and measures

All primary and secondary outcomes were reported at the level of the individual. Specially trained researchers and clinicians measured all outcomes. The primary outcome was the change in aerobic fitness. Secondary outcomes included changes in body composition, BMI, HbA_1c_, lower limb muscle strength, walking speed, balance, flexibility, BP, and lipids. Cardiometabolic parameters were measured during the initial medical visit. Physical fitness and body composition measures were assessed directly in the clinics.

#### 2.4.1 Physical fitness

Physical fitness was assessed by five parameters: aerobic fitness, lower limb muscle strength, walking speed, balance, and flexibility.

Aerobic fitness was evaluated with a maximal graded cycle ergometry test performed by a cardiologist blinded to the other data. Participants started at 20 Watts. Increments of 20 Watts per 2 min were made until exhaustion or until reaching one of the American College of Sports Medicine established criteria for maximal oxygen uptake (23). Heart rate was continuously measured by ECG. Blood pressure and the rate of perceived exertion (24) were assessed at the end of each step. The maximum achieved resistance (Watts) was retained for all calculations.

Lower limb muscle strength was assessed with the Chair Stand Test. Participants sat with arms folded across the chest and with their back against the chair. The patient was instructed to stand up and sit down five times as quickly as possible and the required time (sec) was recorded (25). Walking speed was evaluated using the 10 m walking test; the participant walked at her/his preferred speed and the time (sec) was recorded (26). Balance was evaluated with a single-leg balance test. Participants were instructed to maintain their balance on their preferred leg as long as possible. The test was stopped at 30 seconds and the maximal balance time (sec) was recorded (27). Flexibility was measured using the finger-to-floor distance. After bending forward, the distance (cm) between the tip of the middle finger and the floor was measured (28). All physical fitness tests were performed by one single trained blinded evaluator per centre.

#### 2.4.2 Body composition

During the medical visits, body weight and height were measure barefoot and clothes off. BMI was calculated based on measured height and weight. Waist circumference was measured midway between the iliac crest and the lowest border of the rib cage. Body composition was assessed by bioelectrical impedance using a 4-polar single frequency device (RJL Systems, Model 101A; Detroit, MI, USA). The fat and lean body mass were predicted using the software Bodygram^®^ (Akern Srl., Pontassieve, Florence, Italy).

#### 2.4.3 Cardiometabolic measures

Each centre measured HbA_1c_ and fasting lipid values at the beginning and the end of each program in their local laboratory. HbA_1c_ was measured using high-performance liquid chromatography (HPLC), which is IFCC (International Federation of Clinical Chemistry and Laboratory Medicine) standardized (29). Blood pressure was measured three times during the medical visit while sitting and the mean value of the last two measures was taken.

#### 2.4.4 Confounder variables

Along with age and sex, educational level (EL) was included as a covariate since it’s related to physical fitness in patients with type 2 diabetes (30). Educational level was determined as the highest level of education based on a questionnaire (31).

### 2.5 Statistical analysis

Statistical analyses were conducted using STATA 16 (Stata Corp, College Station, TX, USA). Taking into account a drop-out of 1 centre with an average program size of 10 patients per centre, we assumed that 7 patients per centre would participate in both aerobic fitness tests (due to non-participation, attrition, moving, sickness on the testing day). In case of sickness on the testing day, the stress test (aerobic fitness test) will be repeated on another day. A total number of 10 centres (70 patients per treatment arm) would then provide enough power to detect a true intervention effect of two-thirds of an inter-subject standard deviation at the usual significance level of 0.05 with a probability of 0.8, provided that the standard deviation of the random class effect does not exceed 3% of the inter-subject standard deviation (i.e., corresponding to an intra-class correlation of about 0.03). Therefore, we included 11 treatment centres.

Complete-case analyses were performed according to intention to treat. Descriptive statistics were calculated using mean ± SD for continuous variables, or percentage for categorical variables. The analyses of the primary and secondary outcomes were based on a superiority assumption and presented as mean differences with 95% CI and *P* values for superiority. Student’s t-test for paired samples was used to examine the effectiveness of the DIAfit program on primary and secondary outcomes between baseline and the end of the program. For the primary outcome, 162 patients were analysed and according to the specific outcome measure, 154 to 179 patients were analysed, see **Table 2**.

**Table 2:** Main baseline characteristic of the participants.

Variables	All participants (n= 184)	Standard (n=88)	Alternative (n=96)
Age, mean (SD), years	59.7 (10.2)	59.6 (9.5)	59.7 (10.9)
Women, No. (%)	88 (48%)	36 (41%)	52 (54%)
Obesity prevalence, No. (%)			
*Normal weight*	16 (9%)	6 (7%)	9 (10%)
*Overweight*	48 (28%)	18 (23%)	30 (32%)
*Obesity*	110 (63%)	55 (70%)	55 (58%)
Educational level, No. (%)			
*Low*	69 (44%)	34 (46%)	35 (43%)
*Middle*	51 (33%)	28 (38%)	23 (28%)
*High*	36 (23%)	12 (16%)	24 (29%)
Glucose-lowering medication, No. (%)			
Insulin	70 (40%)	27 (34%)	43 (45%)
GLP1-receptor agonists	39 (22%)	13 (17%)	26 (27%)
Oral antidiabetic agents	148 (85%)	67 (85%)	81 (85%)
Other medication use, No. (%)			
Antihypertensive medications	121 (70%)	58 (73%)	63 (67%)
Lipid-lowering agents	101 (59%)	48 (62%)	53 (56%)
Antiplatelet agents	82 (47%)	39 (49%)	43 (45%)
Diabetes-related parameters, No. (%)			
Diabetes duration, years (SD)	8.9 (7.9)	8.8 (7.9)	9.2 (7.8)
Presence of retinopathy	12 (7%)	5 (7%)	7 (8%)
Presence of nephropathy	14 (9%)	8 (11%)	6 (8%)
Presence of Ischemic cardiac disease	24 (15%)	9 (12%)	15 (17%)
Presence of hypertension	130 (75%)	62 (79%)	68 (73%)
Presence of hyperlipidaemia	128 (75%)	60 (78%)	67 (71%)
Neuropathy: Vibration Threshold (score 0-8)	5.6 (2.2%)	5.7 (2.1%)	5.4 (2.2%)
Hypoglycaemia severe	8 (5%)	5 (7%)	3 (4%)

The presence of complications and comorbidities are obtained based on the information of the referring physician or the patient.

Linear regression analyses were performed to test the difference in outcome at the end of the program between both arms. In the regression models, the dependent variable was represented by baseline to end-of-study change in HbA_1c,_ lower limb muscle strength, balance, flexibility, diastolic BP, LDL, total cholesterol and triglycerides. The predictors were the study arm (=program variant), age, sex, and educational level.

In case of significant association between change in outcome and the centre, mixed model analyses were performed. This was the case for aerobic fitness, walking speed, body weight, BMI, lean body mass, fat body mass, HDL, systolic BP. In the mixed models, the centre was the unit of randomization and change in physical fitness, body composition, and cardiometabolic health outcomes as the dependent variables. The predictors were the study arm, age, sex, and educational level. The objective was to check for each outcome if the coefficient related to the program type is significantly different from 0. Statistical level was set at P<0.05 for all analyses.

To account for missing values, in a secondary analysis all cases were included in the analysis by using multivariate imputation by chained equation (MICE), with the assumption that the data are missing at random (32). Results did not differ between complete-case and multivariate imputation. Therefore, we reported our results based on complete-case analysis.

## 3 Results

**Figure 1:**
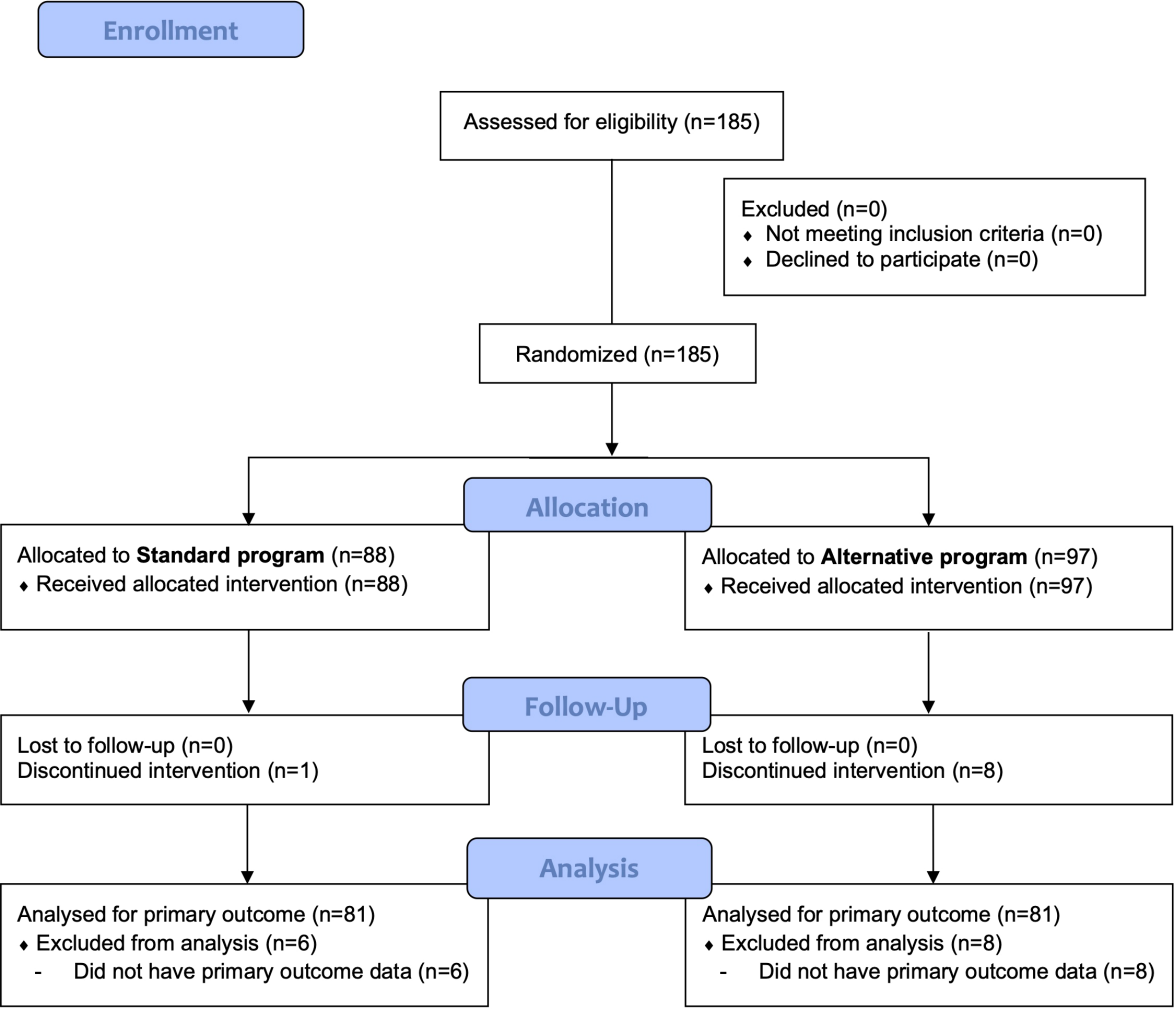
Participant flow chart.

**Figure 1** shows the flow chart of the trial profile. All 185 eligible participants entered the study and were randomly assigned to group (88 in standard group and 97 in alternative group) after stratification.

Baseline characteristics of the study participants are reported in **Table 2**. Mean age was 59.7 ± 10.2 years with a disease duration of 8.9 ± 7.9 years and 52% were men. There were 85% of patients using oral diabetic agents, 22% subcutaneous GLP-1 receptor agonists, and 40% using insulin.

**Table 3** summarizes physical fitness, body composition and cardiometabolic outcomes at baseline and at the end of the DIAfit program and the within-group changes between the two time points. Baseline mean BMI was 32.62 ± 4.98, HbA1c level was 7.28%, and aerobic fitness was 110.95 ± 43.53 Watts.

**Table 3:** Physical fitness, body composition, and cardiometabolic outcomes at baseline and at the end of the DIAfit program of all participants and within-group changes.

	Baseline		After intervention		Effect estimate	
	n	Value (SD)	n	Value (SD)	Mean difference (95% CI)	P value
Physical fitness						
Aerobic fitness (Watts)	162	110.94 (43.53)	107	123.45 (48.43)	12.50 (6.76, 18.25)	**<0.001**
Lower limb strength (sec)	177	13.45 (3.51)	149	10.49 (2.51)	-2.95 (-3.43, -2.48)	**<0.001**
Flexibility (cm)	178	11.94 (9.99)	149	8.49 (8.57)	-3.45 (-4.43, -2.48)	**<0.001**
Balance (sec)	178	20.45 (10.64)	151	22.39 (9.82)	1.94 (0.55, 3.32)	**0.006**
Walking speed (10m, sec)	179	8.21 (1.53)	150	6.94 (1.30)	-1.27 (-1.46, -1.08)	**<0.001**
Anthropometry						
Weight (kg)	179	92.09 (17.38)	143	91.16 (17.78)	-0.93 (-0.43, -1.42)	**<0.001**
BMI (kg/m^2^)	173	32.62 (4.98)	137	32.28 (5.06)	-0.34 (-0.51, -0.16)	**<0.001**
Waist circumference (cm)	157	111.23 (11.55)	127	110.09 (12.45)	-1.14 (-0.32, -1.97)	**0.007**
Body Composition						
Body fat mass (kg)	154	35.34 (10.81)	124	34.02 (9.89)	-1.32 (-2.27, -0.37)	**0.007**
Lean body mass (kg)	154	56.40 (13.42)	124	56.93 (12.76)	0.52 (-0.32, 1.37)	0.22
Cardiometabolic parameters						
HbA1c, (%)	166	7.28 (1.31)	131	7.09 (1.25)	-0.19 (-0.34, -0.05)	**0.009**
Triglycerides (mmol/L)	167	1.78 (0.90)	128	1.80 (1.13)	0.02 (-0.13, 0.17)	0.82
Cholesterol (mmol/L)						
*Total*	168	4.68 (1.13)	129	4.62 (1.03)	-0.06 (-0.20, 0.07)	0.37
*HDL*	168	1.19 (0.38)	128	1.22 (0.36)	0.03 (-0.01, 0.06)	0.11
*LDL*	162	2.64 (0.92)	121	2.63 (0.94)	-0.01 (-0.12, 0.11)	0.89
BP (mmHg)						
*Systolic*	174	133 (15.4)	128	130 (14.8)	-3 (-6.1, -0.8)	**0.01**
*Diastolic*	174	82 (9.9)	127	79 (8.9)	-3 (-4.6, -1.5)	**<0.001**

SD, standard deviation; CI, confidence interval; BMI, body mass index; HbA_1c_, glycosylated haemoglobin; HDL, high density lipoprotein; LDL, low density lipoprotein; BP, blood pressure. Significant differences are shown in bold.

### 3.1 Physical fitness

Compared to baseline, aerobic fitness increased by 12.5 Watts (95% CI 6.76 to 18.25; p<0.001). This corresponds to a mean improvement of 11%. Also, at the end of the program participants needed 2.95sec (95% CI 2.48 to 3.43; p<0.001) less for the lower limb strength test (=increased strength), increased their flexibility by 3.45cm (95% CI -4.43 to -2.48; p<0.001), could stand on one leg for 1.94sec (95% CI 0.55 to 3.32; p=0.006) longer, and needed 1.27sec (95% CI -1.46 to -1.08; p<0.001) less for the 10m walking speed test. In summary, there was a mean improvement of 10-29% in the secondary physical fitness outcomes.

### 3.2 Body composition

Body weight decreased by 0.93kg (95% CI -0.43 to -1.42; p<0.001), BMI decreased by 0.34 kg/m^2^ (95% CI -0.51 to -0.16; p<0.001) and waist circumference decreased by 1.14cm (95% CI -0.32 to -1.97; p=0.007). Body fat mass decreased by 1.32kg (95% CI -2.27 to -0.37; p=0.007) between baseline and the end of the program, while there was no difference in lean body mass.

### 3.3 Cardiometabolic measures

Between baseline and the end of the program, HbA_1c_ decreased by 0.19% (95% CI -0.34 to -0.05; p=0.009) and systolic and diastolic BP decreased by 3 mmHg (95% CI-6.1 to -0.8; p=0.01) and 3 mmHg (95% CI -4.6 to -1.5; p<0.01), respectively. There were no effects on triglycerides and cholesterol levels (p>0.11).

**Table 4** shows the differences in physical fitness, body composition, and cardiometabolic outcomes in the standard and alternative program and the between-group differences.

**Table 4:** Differences in physical fitness, body composition, and cardiometabolic outcomes in the standard and alternative program and between-group differences.

	Standard program (n=88)		Alternative program (n=97)		Adjusted net difference in change between groups	
	Mean difference (95% CI)	P value	Mean difference (95% CI)	P value	Mean difference (95% CI)	P value
**Total sessions attended (no)**	30.6 (4.11)		28.9 (5.08)		1.70 (-0.22, 3.62)	0.08
Physical fitness						
Aerobic fitness (Watts)	9.27 (3.70, 14.84)	**0.002**	16.20 (5.52, 26.89)	**0.004**	-0.80 (-12.22, 10.61)	0.89‡
Lower limb strength (sec)	-3.01 (-3.73, -2.30)	**<0.001**	-2.88 (-3.51, -2.26)	**<0.001**	0.15 (-0.87, 1.17)	0.77
Flexibility (cm)	-3.98 (-5.01, -2.96)	**<0.001**	-2.85 (-4.61, -1.10)	**0.002**	1.26 (-0.85, 3.38)	0.24
Balance (sec)	1.08 (-1.09, 3.25)	0.33	2.89 (1.21, 4.57)	**0.001**	2.46 (-0.38, 5.31)	0.09
Walking speed (10m, sec)	-1.31 (-1.55, -1.08)	**<0.001**	-1.22 (-1.53, -0.91)	**<0.001**	-0.16 (-0.52, 0.20)	0.39‡
Anthropometry						
Weight (kg)	-1.36 (-2.03, -0.70)	**<0.001**	-0.40 (-1.13, 0.33)	0.28	0.97 (0.04, 1.91)	**0.04**‡
BMI (kg/m^2^)	-0.49 (-0.74, -0.24)	**0.001**	-0.16 (-0.41, 0.08)	0.18	0.33 (0.02, 0.65)	**0.04**‡
Waist circumference (cm)	-1.49 (-2.29, -0.68)	**<0.001**	-0.75 (-2.29, 0.79),	0.33	0.82 (-0.75, 2.39)	0.31‡
Body Composition						
Body fat mass (kg)	-1.82 (-3.41, -0.23)	**0.03**	-0.79 (-1.82, 0.23)	0.13	1.41 (-0.33, 3.15)	0.11‡
Lean body mass (kg)	0.51 (-1.03, 2.05)	0.51	0.54 (-0.14, 1.23)	0.12	-0.32 (-1.89, 1.25)	0.69‡
Cardiometabolic parameters						
HbA1c, (%)	-0.26 (-0.46, -0.05)	**0.02**	-0.13 (-0.33, 0.08)	0.22	0.19 (-0.11, 0.50)	0.22
Triglycerides (mmol/L)	-0.01 (-0.19, 0.18)	0.95	0.04 (-0.20, 0.29)	0.74	0.52 (-0.25, 0.40)	0.66
Cholesterol (mmol/L)						
*Total*	-0.09 (-0.25, 0.08)	0.28	-0.03 (-0.25, 0.18)	0.76	0.14 (-0.14, 0.42)	0.33
*HDL*	0.01 (-0.04, 0.06)	0.64	0.04 (-0.002, 0.09)	0.06	0.03 (-0.04, 0.10)	0.34‡
*LDL*	-0.06 (-0.22, 0.09)	0.42	0.05 (-0.12, 0.22)	0.54	0.20 (-0.03, 0.44)	0.09
Blood pressure (mmHg)						
*Systolic*	-4.1 (-7.5, -0.8)	**0.02**	-2.6 (-6.7, 1.5)	0.21	1.3 (-4.2, 6.8)	0.65‡
*Diastolic*	-2.4 (-4.3, -0.5)	**0.02**	-3.9 (-6.5, -1.2)	**0.005**	-1.6 (-5.1, 1.8)	0.35

CI, confidence interval; BMI, body mass index; HbA_1c_, glycosylated haemoglobin; HDL, high density lipoprotein; LDL, low density lipoprotein; BP, blood pressure.

Differences between groups are shown comparing the alternative vs the standard program adjusted for age; sex; and educational level.

‡The ANOVA analyses revealed that the centre (cluster) was associated with the outcome; so for these outcomes mixed models were used. For all other outcomes; linear regression analyses were used.Significant differences are shown in bold.

Except for body weight, there were no significant differences in all outcomes between the standard and the alternative programs (p>0.05). The difference favoured the standard group, with a more pronounced weight reduction of -0.94kg (95% CI -0.05 to -1.83; p=0.04). For weight metrics, the effects remained the same after adjusting for weight status at baseline (data not shown) when comparing both intervention programs.

Throughout the 36 sessions, the average exercise training attendance was 30.6 sessions (85%) for the standard program and 28.9 sessions (80%) for the alterative program (1.7; 95% CI -0.22-3.62, p=0.08).

## 4 Discussion

This pragmatic randomized trial demonstrated the effectiveness of a real-life nationwide PA group program (DIAfit) in improving aerobic fitness and other physical fitness outcomes such as strength, flexibility, balance and walking speed by 10-29%. Although to a lesser extent, the program also improved body weight, body composition, HbA_1c_, and BP. Our results also showed that both a standard “classical” approach to exercise frequency as well as an alternative “feasible” approach led to similarly improved health outcomes with an additional benefit of the standard program on weight reduction.

This study showed important findings regarding translating evidence from highly specialized research centres into real-life clinical practice. Indeed, all patients enrolled in the clinical DIAfit program were eligible and agreed to participate in the study. The program took mostly place in small regional hospitals, but also in private practices or university hospitals. The DIAfit program was delivered by regular staff with no specific experience. All of which increased the generalizability of our findings. Furthermore, patients in our study were of similar age or older than those in previous studies. They had a mean baseline HbA1c of 7.3% which is representative of the glycaemic control observed in larger community-based cohorts of patients with diabetes in Switzerland (33). There was also a higher prevalence of insulin use (40%) in our study than in most previous studies (11, 16, 34). It is encouraging to observe that the improvements in physical fitness measures, anthropometry, body composition and most cardiometabolic outcomes are similar to those observed in specialized centres.

We evaluated a comprehensive range of physical fitness outcomes. The primary outcome, aerobic fitness, improved by 11%. Similar results were reported in a previous meta-analysis that demonstrated an 11.8% increase in VO_2max_ after intervention (10). Aerobic fitness is an important independent modifiable risk factor and even modest improvements are likely to have beneficial consequences on morbidity and mortality (22, 35–37). In our study, other fitness measures such as muscular strength, flexibility, balance, and walking speed improved as well, with improvements ranging between 10-15% (for balance and walking speed) to 22-29% (for muscle strength and flexibility). These findings are relevant since muscular strength and walking speed are associated with lower risk of cardiovascular disease and all-cause mortality among middle-aged adults and type 2 diabetes patients (2, 4, 38–42). Furthermore, improvements in muscular strength and walking speed observed in our study could contribute to regaining muscle strength and prevention of frailty in this population. As many older adults with diabetes have peripheral neuropathy and vibration examination showed a modest neuropathy in our population, encouraging them to perform balance and flexibility exercises 2-3 times/week is crucial to prevent falls and reduce injuries (4). Our results support the fact that flexibility and balance training are an integral part of the current exercise guidelines, and it’s particularly important for older adults with diabetes to maintain range of motion, strength, and balance (6).

The DIAfit program was associated with a reduction of a 0.19% in HbA_1c_. This reduction is less pronounced than what was observed in a meta-analysis of structured exercise programs (0.5%) (8). These differences can be explained by the fact that our patients had higher insulin use and often ate to prevent or compensate for hypoglycaemia. The non-selectivity of our program might have also contributed to these differences. The observed BP reduction is similar to previous PA interventions (11, 16, 34). On the other hand, the DIAfit program did not have a significant effect on plasma triglycerides or cholesterol level. To achieve greater changes, higher volumes or intensities of exercise might be necessary (43).

The DIAfit program was effective in improving body composition. The observed mean reduction in weight of 0.93 kg and waist circumference of 1.14 cm are similar to results found in some structured PA programs of similar duration without any dietary intervention and demonstrates the effects that can be achieved with this intensity and volume of PA (8, 9, 13). The waist circumference, but not weight reduction observed in our study is similar to the changes observed in one of the largest and longest randomized trial (Look AHEAD) assessing the impact of a combined very intensive lifestyle intervention on weight loss, 8 years after the intervention (44). The improvements in body fat observed in our study were similar to those observed in other 3 months (11) and 9 months intervention studies (34). The level of waist circumference and body fat reduction found in our study may have a meaningful clinical effect. Our results and the Look AHEAD trial provided strong evidence of profound health benefits from lifestyle intervention.

In this study, we also compared two program variants and found similar results for physical fitness, body composition, and cardiometabolic parameters in both programs although effect sizes of changes were larger in the standard program regarding anthropometric parameters, body fat mass, HbA_1c_ and blood pressure, and in some circumstances the changes were only significant in the standard program. However, significant difference between both programs were only found for weight and BMI with a more marked weight reduction in the standard program. Reasons for differences in weight reduction could lie in a more short-lasting willpower regarding concomitant changes in nutrition or in overcompensating hypoglycaemia. Physical activity attendance did not differ between the standard and the alternative program. The current results demonstrate that the alternative “feasible” approach is not necessarily more feasible for all patients, and the standard “physiological” approach showed some benefits regarding weight. Based on these results, for patients who have no preferences at all, we would suggest the standard program. However, according to patient preferences, both type of programs could represent an almost equally effective and feasible strategies for improving health in patients with type 2 diabetes. Our results based on real-life observation provide evidence regarding the possible flexibility when designing future exercise programs for this population. To achieve therapeutic goals in type 2 diabetes patients, it is important to consider optimal type, frequency and duration of exercise when designing PA programs to meet the specific needs of each individual. To our knowledge, effects of different exercise frequencies on multiple health outcomes have not been compared so far. Previous intervention studies in patients with type 2 diabetes conducted in specialized centres using two sessions per week demonstrated improvements similar to our study in aerobic fitness, muscular strength, and body composition after 4-12 months intervention (10, 16, 18, 20). Other studies using also 2x/week structured PA for 5 to 12 months have observed similar reductions in BP (3-5mmHg) and slightly higher reductions of HbA_1c_ (0.10-0.45%) compared to our findings (16, 18, 19). In contrast, some studies did not observe any changes in HbA_1c_, BP, body weight after intervention (17, 20). These differences can be explained by the fact that intervention duration, intensity of exercise programs and sample size differed between studies. Furthermore, there is still insufficient evidence on the exact intensity, volume, and duration of exercise to provide health benefits in type 2 patients (12). Our results are relevant for patients with time restriction or other obstacles since 1-2 PA sessions/week might be an effective strategy leading to beneficial health outcomes, especially in fitness measures. Our findings showed that progressively increasing exercise frequency to meet individual needs improved aerobic fitness, strength, flexibility, walking speed and cardiometabolic health to a similar extent than starting 3x/week from the very beginning when the total number of sessions is the same. In real-life daily practice, one size fits all approach rarely exists. Patients may want to establish their personal objectives and gradually engage patients in increasing PA levels into an already busy schedule. As stated by the American Diabetes Association in 2023, recommendations should be tailored to meet the specific needs of each individual in order to promote the adoption and maintenance of lifetime PA.

Strengths of the current study included the pragmatic nature of a nation-wide PA program which demonstrated the benefits and relevance of structured PA for patients with type 2 diabetes in real-world conditions integrated in routine care. The multicentre design in different settings increased the external validity since the results were observed in different clinical settings. Furthermore, we included a broad range of outcome measures including extensive physical fitness measures relevant for various health outcomes such aerobic fitness, muscular strength, flexibility, balance, and walking speed. And lastly, the comparison of different forms of exercise frequency is not only novel, but also pertinent for clinical practice. Some limitations should be noted. The most important one being the lack of a control group for our first aim. However, participating health care providers and cantonal agencies did not approve the idea of a waiting list which would not let benefit all patients at the time when they are motivated or at least agree to participate in a PA program. All providers were told not to adapt the medical treatment during the testing time period, but we did not control for any potential changes in medication intake. In this real-life trial, small differences between centres were observed in several outcomes such as aerobic fitness, walking speed, anthropometry, body composition, and some cardiometabolic parameters, see **Table 3**, which may hint to the quality of a specific centre in delivering the program and affecting the outcomes. This could attenuate the external validity of our findings. Also, in our study there was an attrition of 12.5% for the primary outcome which, although not very high for a pragmatic lifestyle trial in patients with diabetes, may be a source of bias. Furthermore, the study was conducted around 10 years ago. Unfortunately, we did not collect long-term follow-up data on physical fitness, cardiometabolic health and body composition. Since the implementation of the clinical program was sponsored by the state for only two years, we unfortunately did not have the personal and infrastructural and financial resources to conduct the follow-up.

## 5 Conclusion

The national DIAfit PA group program was effective and deliverable in real-world clinical settings for promoting PA and improving important health parameters such as physical fitness, cardiometabolic health, and body composition in patients with type 2 diabetes. The alternative program of one-to-twice weekly supervised combined aerobic and resistance exercise had similar benefits compared to the classical program and can serve as a useful example for implementing a site-tailored PA intervention.

## Data availability statement

Part of the original contributions presented in the study are included in the article/supplementary material. Further inquiries can be directed to the corresponding author.

## Ethics statement

The studies involving human participants were reviewed and approved by Lausanne University Hospitals Ethical Committee. The patients/participants provided their written informed consent to participate in this study.

## Author contributions

JP and OG with the assitance of HD, MC, DD, SB and DS were responsable for the study design and all authors helped data collection. J-BR, AA and JP were responsible for the statistical analysis and AA and NJ helped with data management. AA and JP wrote the manuscript draft. All authors contributed to the article ad approved the submitted version.
